# Supplementary material for: Re-Emerged Genotype IV of Japanese Encephalitis Virus Is the Youngest Virus in Evolution
Source: Viruses. 2023 Feb 24;15(3):626. doi: 10.3390/v15030626 (PMC10054483; doi:10.3390/v15030626)
Supplement: Supplementary file 1 [file viruses-15-00626-s001.zip › Figure S2:Comparison of C protein segments of different JEV genotypes..pdf]

```
                                          *       120         *       140         *       160         *       180         *       200
K94P05                         : KQNKRGGNERSIMWLASLAIVTAYAGAMKLSNFQGKLLMTINNTDIADVIVIPTSKGENRCWVRAIDVGYMCEDTITYECPKLAVGNDPEDVDCWCDNQE : 200
FU                             : .........G.........V.M.C................V..........................................TT............... : 200
JaOArS982                      : .........G.........V.I.............................................................TM............... : 200
VN-113                         : ........GGTTL.FM..T.AAVCV..L...........AV...........................................P..............D : 200
JKT6468                        : ........GGTTL.FM..T.AAVCV..L...........AV..........................................TP....Q.......H.. : 200
JEV/sw/Bali/93/2017            : ........GGTVL..M..T.AGVSV..L.....................................................R..P..............D : 200
Bali-2019                      : ........GGTVL..M..T.AGISV..L.....................................................R..P..............D : 200
19CxBa-83-Cv                   : ........GGTVL..M..T.AGVSV..L.....................................................R..P..............D : 200
JEV/Human/NT-Tiwi-Islands/2021 : ........GGTVL..M..T.AAVSV..L..........................................T..........R..P..............D : 200
JEV/sw-22-00722-11/Qld/2022    : ........GGTVL..M..T.AAVSV..L..........................................T..........R..P..............D : 200
Muar                           : .......SNGT.I.MIG.AVVF.TVS.V..................V....T......T...........H.............DA......I.....K.A : 200
```

Figure S2: Comparison of C protein segments of different JEV genotypes. All GⅣ strains are represented with green shades, and the name of old sublineage isolates are different with italics. The underlined part of the sequence is the hydrophobic transmembrane domain of C protein. The bases marked with blue shades representing the predicted signal enzyme/protease cleavage motif, and the bases marked with red shades representing the predicted N-linked glycosylation sites.
