# Supplementary material for: Re-Emerged Genotype IV of Japanese Encephalitis Virus Is the Youngest Virus in Evolution
Source: Viruses. 2023 Feb 24;15(3):626. doi: 10.3390/v15030626 (PMC10054483; doi:10.3390/v15030626)
Supplement: Supplementary file 1 [file viruses-15-00626-s001.zip › Table S1:The average nucleotide and amino acid identities of the JEVs’ whole genome.pdf]

Table S1: The average nucleotide and amino acid identities of the JEVs' whole genome

Within genotypes (nt %, aa%)													
Genotype	5'UTR	C	PrM	E	NS1	NS2A	NS2B	NS3	NS4A	NS4B	NS5	3'UTR	Complete
Ⅰ	99.0	97.8(98.2)	96.7(98.0)	96.8(99.2)	97.6(99.5)	97.0(98.7)	96.8(99.5)	97.3(99.5)	97.1(99.5)	97.7(99.7)	97.2(99.3)	96.1	97.1(99.2)
Ⅱ	100	100(100)	100(100)	100(100)	100(100)	100(100)	100(100)	100(100)	100(100)	100(100)	100(100)	100	100(100)
Ⅲ	99.1	97.6(98.1)	97.1(98.5)	97.3(98.7)	97.6(99.0)	97.0(98.7)	97.4(99.2)	97.4(99.3)	97.0(99.2)	97.0(99.2)	97.5(99.2)	96.9	97.4(99.0)
Ⅳ	**98.3**	**97.3(95.4)**	**95.3(96.0)**	**96.6(98.6)**	**97.5(99.6)**	**97.0(99.3)**	**97.2(99.8)**	**96.4(99.1)**	**96.7(99.8)**	**96.2(100)**	**96.8(99.2)**	**91.5**	**96.4(98.9)**
Ⅴ	98.8	95.4(95.8)	96.2(99.7)	93.9 (99.2)	94.3(99.0)	93.3(99.2)	94.1(99.3)	94.4(99.1)	92.9(97.7)	94.2(100)	94.3(99.0)	95.4	94.3(98.9)

Table S1: The average nucleotide and amino acid identities of the JEVs' whole genome. The table shows the nucleotide and amino acid identities of structural protein, non-structural protein, non-coding region and whole genome of JEV in genotypes. The amino acid identities are shown in brackets. There is only one isolate of GII, which cannot display more information. GIV JEV is our most concerned in this study, which is marked by bold.
