# Supplementary material for: Re-Emerged Genotype IV of Japanese Encephalitis Virus Is the Youngest Virus in Evolution
Source: Viruses. 2023 Feb 24;15(3):626. doi: 10.3390/v15030626 (PMC10054483; doi:10.3390/v15030626)
Supplement: Supplementary file 1 [file viruses-15-00626-s001.zip › Table S2: Amino acid mutations in structural and non-structural proteins of JEV GIV isolates.pdf]

Table S2: Amino acid mutations in structural and non-structural proteins of JEV GⅣ isolates

No.	Protein	Position of mutation	Standard strain	GⅣ						
				Old sublineage		Emerging sublineage				
						Indonesia branch			Australia branch	
			P3	Jkt6468	VN_113	Bali 93	Bali-2019	CxBa_83-CV	Nt-tiwi	Sw-22-0072
1.	C	6	Gly	Arg	Gly	Gly	Gly	Gly	Gly	Gly
2.		10	Lys	Ile	Lys	Lys	Lys	Lys	Lys	Lys
3.		15	Asn	Tyr	Asn	Asn	Asn	Asn	Asn	Asn
4.		41	Arg	Ile	Arg	Arg	Arg	Arg	Arg	Arg
5.		50	Phe	Leu	Leu	Leu	Leu	Leu	Leu	Leu
6.		52	Thr	Ser	Ala	Ala	Ala	Ala	Ala	Ala
7.		60	Ala	Ser	Ala	Ala	Ala	Ala	Ala	Ala
8.		75	Ser	Ser	Ser	Gly	Gly	Gly	Gly	Gly
9.		109	Glu	Gly	Gly	Gly	Gly	Gly	Gly	Gly
10.		111	Ser	Thr	Thr	Thr	Thr	Thr	Thr	Thr
11.		112	Ile	Thr	Thr	Val	Val	Val	Val	Val
12.		113	Met	Leu	Leu	Leu	Leu	Leu	Leu	Leu
13.		115	Leu	Phe	Phe	Leu	Leu	Leu	Leu	Leu
14.		116	Ala	Met	Met	Met	Met	Met	Met	Met
15.		119	Ala	Thr	Thr	Thr	Thr	Thr	Thr	Thr
16.		120	Ala	Ile	Ile	Ile	Ile	Ile	Ile	Ile
17.		121	Val	Ala	Ala	Ala	Ala	Ala	Ala	Ala

18.		122	Ile	Ala	Ala	Gly	Gly	Gly	Ala	Ala
19.		123	Ala	Val	Val	Val	Ile	Val	Val	Val
20.		124	Cys	Cys	Cys	Ser	Ser	Ser	Ser	Ser
21.		125	Ala	Val	Val	Val	Val	Val	Val	Val
1.	PrM	1	Met	Leu	Leu	Leu	Leu	Leu	Leu	Leu
2.		2	Arg	Lys	Lys	Lys	Lys	Lys	Lys	Lys
3.		13	Thr	Ala	Ala	Thr	Thr	Thr	Thr	Thr
4.		14	Ile	Val	Val	Ile	Ile	Ile	Ile	Ile
5.		44	Met	Met	Met	Met	Met	Met	Thr	Thr
6.		55	Lys	Lys	Lys	Arg	Arg	Arg	Arg	Arg
7.		57	Thr	Thr	Ala	Ala	Ala	Ala	Ala	Ala
8.		58	Met	Pro	Pro	Pro	Pro	Pro	Pro	Pro
9.		63	Glu	Gln	Glu	Glu	Glu	Glu	Glu	Glu
10.		71	Asn	His	Asn	Asn	Asn	Asn	Asn	Asn
11.		73	Glu	Glu	Asp	Asp	Asp	Asp	Asp	Asp
12.		85	Arg	Ser	Arg	Arg	Arg	Arg	Arg	Arg
13.		88	Lys	Gln	Lys	Lys	Lys	Lys	Lys	Lys
14.		89	Arg	Thr	Arg	Arg	Arg	Arg	Arg	Arg
15.		97	Gln	His	Gln	Gln	Gln	Gln	Gln	Gln
16.		107	Lys	Lys	Lys	Lys	Lys	Arg	Lys	Lys
17.		113	Asp	Asn	Asp	Asp	Asp	Asp	Asp	Asp
18.		122	Met	Val	Met	Met	Met	Met	Met	Met
19.		130	Ile	Val	Ile	Ile	Ile	Ile	Ile	Ile
20.		139	Ala	Ala	Val	Val	Val	Val	Val	Val
21.		140	Val	Val	Ala	Ala	Ala	Ala	Ala	Ala
22.		149	Asn	Ser	Ser	Ser	Ser	Ser	Ser	Ser

23.		151	Gln	Pro	Gln	Gln	Gln	Gln	Gln	Gln
24.		153	Val	Val	Val	Val	Val	Val	Met	Met
1.	E	15	Ala	Val	Val	Val	Val	Val	Val	Val
2.		36	Asn	Asn	Asn	His	His	His	His	His
3.		38	Lys	Arg	Arg	Arg	Arg	Arg	Arg	Arg
4.		46	Thr	Ile	Ile	Ile	Ile	Val	Ile	Ile
5.		76	Met	Thr	Thr	Thr	Thr	Thr	Thr	Thr
6.		83	Glu	Glu	Glu	Asp	Asp	Asp	Glu	Glu
7.		89	Ser	Ser	Ser	Asn	Asn	Asn	Ser	Ser
8.		126	Ile	Thr	Thr	Thr	Thr	Thr	Ile	Ile
9.		128	Arg	Lys	Lys	Lys	Lys	Lys	Lys	Lys
10.		129	Ala	Thr	Thr	Thr	Thr	Thr	Thr	Thr
11.		141	Ile	Ile	Ile	Ile	Ile	Ile	Val	Val
12.		156	Ser	Thr	Thr	Thr	Thr	Thr	Thr	Thr
13.		159	Val	Ile	Ile	Ile	Ile	Ile	Ile	Ile
14.		169	Val	Ile	Ile	Ile	Ile	Ile	Ile	Ile
15.		194	Ser	Ser	Ser	Ser	Ser	Ser	Asn	Asn
16.		209	Arg	Lys	Lys	Lys	Lys	Lys	Lys	Lys
17.		227	Pro	Ser	Ser	Ser	Ser	Ser	Ser	Ser
18.		228	Pro	Ser	Ser	Ser	Ser	Ala	Ser	Ser
19.		260	Gly	Arg	Gly	Gly	Gly	Gly	Gly	Gly
20.		261	Gly	Ala	Ala	Ala	Ala	Ala	Ala	Ala
21.		295	Ala	Ala	Ala	Thr	Thr	Thr	Thr	Thr
22.		306	Gly	Glu	Glu	Glu	Glu	Glu	Glu	Glu
23.		327	Ser	Leu	Leu	Gln	Gln	Gln	Gln	Gln
24.		351	Ala	Val	Val	Val	Val	Val	Val	Val

25.		366	Ala	Ser	Ser	Ser	Ser	Ser	Ser	Ser
26.		370	Lys	Gln	Lys	Lys	Lys	Lys	Lys	Lys
27.		388	Glu	Gly	Gly	Gly	Gly	Gly	Gly	Gly
28.		399	Ala	Pro	Pro	Pro	Pro	Pro	Pro	Pro
29.		408	Leu	Ser	Ser	Ser	Ser	Ser	Ser	Ser
30.		466	Ala	Val	Val	Ala	Ala	Ala	Ala	Ala
31.		473	Val	Ile	Ile	Val	Val	Val	Ile	Ile
32.		482	Leu	Met	Met	Met	Met	Met	Val	Val
33.		486	Ala	Val	Val	Val	Val	Val	Val	Val
34.		490	Val	Thr	Thr	Thr	Thr	Thr	Thr	Thr
35.		492	Val	Leu	Leu	Leu	Leu	Leu	Leu	Leu
1.	NS1	51	Lys	Met	Met	Met	Met	Met	Met	Met
2.		54	Val	Ile	Ile	Ile	Ile	Ile	Ile	Ile
3.		95	Ser	Ser	Ser	Ser	Ser	Ser	Pro	Pro
4.		175	Ser	Asn	Asn	Asn	Asn	Asn	Asn	Asn
5.		182	Ala	Thr	Thr	Thr	Thr	Thr	Thr	Thr
6.		188	Val	Ile	Ile	Ile	Ile	Ile	Ile	Ile
7.		205	Arg	His	His	His	His	His	His	His
8.		206	Tyr	Leu	Leu	Leu	Leu	Leu	Leu	Leu
9.		220	Val	Ile	Ile	Ile	Ile	Ile	Ile	Ile
10.		271	Asn	Asp	Asp	Asp	Asp	Asp	Asp	Asp
11.		317	Ser	Thr	Thr	Thr	Thr	Thr	Thr	Thr
12.		326	Glu	Gly	Gly	Gly	Gly	Gly	Gly	Gly
13.		327	Asn	Ser	Ser	Ser	Ser	Ser	Ser	Ser
14.		335	Ile	Val	Val	Val	Val	Val	Val	Val
15.		339	Arg	Lys	Lys	Lys	Lys	Lys	Lys	Lys

16.		343	Thr	Ala	Ala	Ala	Ala	Ala	Ala	Ala
17.		350	Val	Ala	Ala	Val	Val	Val	Val	Val
18.		354	Asn	Ser	Ser	Ser	Ser	Ser	Ser	Ser
19.		358	Val	Ile	Ile	Ile	Ile	Ile	Ile	Ile
20.		386	Ile	Val	Val	Val	Val	Val	Val	Val
21.		407	Ala	Ala	Ala	Val	Ala	Ala	Ala	Ala
1.	NS2A	7	Ser	Asn	Asn	Asn	Asn	Asn	Asn	Asn
2.		29	Met	Ala	Ala	Ala	Ala	Ala	Ala	Ala
3.		56	Val	Ala	Ala	Ala	Ala	Ala	Ala	Ala
4.		65	Ile	Met	Met	Met	Met	Met	Met	Met
5.		71	Ile	Ile	Ile	Ile	Ile	Ile	Thr	Ile
6.		76	Val	Ile	Ile	Ile	Ile	Ile	Ile	Ile
7.		77	Arg	Lys	Lys	Lys	Lys	Lys	Lys	Lys
8.		86	Ser	Thr	Thr	Thr	Thr	Thr	Thr	Thr
9.		87	Val	Ile	Ile	Ile	Ile	Ile	Ile	Ile
10.		91	Val	Leu	Leu	Leu	Leu	Leu	Leu	Leu
11.		97	Thr	Ala	Ala	Ala	Ala	Val	Ala	Ala
12.		113	Val	Ile	Ile	Ile	Ile	Ile	Ile	Ile
13.		116	Ile	Val	Val	Val	Val	Val	Val	Val
14.		121	His	Gln	Gln	Gln	Gln	Gln	Gln	Gln
15.		124	Lys	Arg	Arg	Arg	Arg	Arg	Arg	Arg
16.		125	Lys	Arg	Arg	Lys	Lys	Lys	Arg	Arg
1.	NS2B	56	Gln	Arg	Arg	Arg	Arg	Arg	Arg	Arg
2.		88	Ile	Ile	Ile	Ile	Ile	Val	Ile	Ile
1.	NS3	14	Ser	Ala	Ala	Thr	Thr	Thr	Thr	Thr
2.		44	Asn	Ser	Ser	Gly	Gly	Gly	Gly	Gly

3.		92	Thr	Thr	Thr	Lys	Lys	Lys	Lys	Lys
4.		107	Val	Thr	Thr	Ile	Ile	Ile	Ile	Ile
5.		117	Arg	Cys	Cys	Cys	Cys	Cys	Cys	Cys
6.		169	Asp	Asp	Asp	Glu	Glu	Glu	Glu	Glu
7.		175	Val	Ile	Ile	Ile	Ile	Ile	Ile	Ile
8.		180	Thr	Asn	Asn	Asn	Asn	Asn	Asn	Asn
9.		182	Asn	Ser	Ser	Ser	Ser	Ser	Ser	Ser
10.		185	Arg	Lys	Lys	Lys	Lys	Lys	Lys	Lys
11.		210	Lys	Arg	Arg	Arg	Arg	Arg	Arg	Arg
12.		253	Gln	Gln	Gln	Lys	Gln	Gln	Gln	Gln
13.		294	Ser	Gly	Ser	Ser	Ser	Ser	Ser	Ser
14.		340	Ile	Val	Val	Ile	Ile	Ile	Val	Val
15.		374	Met	Val	Val	Val	Val	Val	Val	Val
16.		382	Lys	Lys	Lys	Arg	Arg	Arg	Arg	Arg
17.		436	Glu	Glu	Glu	Glu	Glu	Glu	Gly	Glu
18.		492	Lys	Lys	Lys	Lys	Lys	Lys	Lys	Arg
19.		495	Met	Leu	Leu	Leu	Leu	Leu	Leu	Leu
20.		518	Ser	Phe	Phe	Phe	Phe	Phe	Phe	Phe
21.		586	Met	Thr	Thr	Met	Met	Met	Met	Met
22.		591	Ile	Val	Val	Ile	Ile	Ile	Ile	Ile
1.	NS4A	3	Val	Ile	Ile	Ile	Ile	Ile	Ile	Ile
2.		5	Phe	Phe	Phe	Phe	Phe	Phe	Leu	Leu
3.		17	Met	Ala	Ala	Ala	Ala	Ala	Ala	Ala
4.		35	Lys	Arg	Arg	Arg	Arg	Arg	Arg	Arg
5.		61	Thr	Ala	Ala	Ala	Ala	Ala	Ala	Ala
6.		72	Met	Lys	Met	Met	Met	Met	Met	Met

7.		88	Thr	Val	Val	Val	Val	Val	Val	Val
8.		100	Pro	Ser	Ser	Ser	Ser	Ser	Ser	Ser
9.		160	Thr	Ala	Ala	Ala	Ala	Ala	Ala	Ala
10.		161	Gln	Pro	Pro	Pro	Pro	Pro	Pro	Pro
11.		162	Ala	Val	Val	Val	Val	Val	Val	Val
12.		165	Leu	Met	Met	Met	Met	Met	Met	Met
13.		180	Ser	Gly	Gly	Gly	Gly	Gly	Gly	Gly
14.		208	Leu	Ile	Ile	Ile	Ile	Ile	Ile	Ile
15.		264	Met	Val	Val	Val	Val	Val	Val	Val
16.		267	Ala	Thr	Thr	Thr	Thr	Thr	Thr	Thr
1.	NS5	15	Lys	Arg	Arg	Arg	Arg	Arg	Arg	Arg
2.		33	Ile	Ile	Ile	Ile	Ile	Ile	Thr	Thr
3.		49	Ile	Lys	Lys	Lys	Lys	Lys	Lys	Lys
4.		72	Ser	Ser	Ser	Ser	Ser	Ser	Thr	Thr
5.		78	Ile	Val	Val	Ile	Ile	Ile	Ile	Ile
6.		135	Lys	Arg	Arg	Arg	Arg	Arg	Arg	Arg
7.		144	Phe	Leu	Leu	Leu	Leu	Leu	Leu	Leu
8.		275	Asn	Asp	Asp	Asp	Asp	Asp	Asp	Asp
9.		277	Glu	Gly	Gly	Gly	Gly	Gly	Gly	Gly
10.		283	Ile	Ile	Ile	Val	Ile	Ile	Ile	Ile
11.		284	Gln	Gln	Gln	Gln	Gln	Gln	Glu	Glu
12.		292	Thr	Thr	Thr	Thr	Thr	Ala	Thr	Thr
13.		296	Lys	Arg	Arg	Arg	Arg	Arg	Arg	Arg
14.		370	Ala	Pro	Pro	Ser	Ser	Ser	Ser	Ser
15.		372	Ala	Val	Val	Val	Val	Val	Val	Val
16.		386	Tyr	Tyr	Tyr	Tyr	Tyr	Tyr	His	His

17.	390	Glu	Glu	Glu	Glu	Glu	Glu	Glu	Glu
18.	429	Asp	Gly	Gly	Asp	Asp	Asp	Gly	Gly
19.	439	Glu	Val	Val	Val	Val	Val	Val	Val
20.	452	Arg	Cys	Cys	Cys	Cys	Cys	Cys	Cys
21.	453	Ile	Val	Val	Val	Val	Val	Val	Val
22.	455	Asn	His	Asn	Asn	Asn	Asn	Asn	Asn
23.	503	Glu	Arg	Arg	Arg	Arg	Arg	Arg	Arg
24.	526	Gly	Arg	Arg	Arg	Arg	Arg	Arg	Arg
25.	528	Gln	Glu	Glu	Glu	Glu	Glu	Glu	Glu
26.	546	Arg	Lys	Lys	Lys	Lys	Lys	Lys	Lys
27.	576	Arg	Lys	Lys	Lys	Lys	Lys	Lys	Lys
28.	586	Ala	Ser	Ser	Ser	Ser	Ser	Ser	Ser
29.	587	Ala	Thr	Thr	Thr	Thr	Thr	Thr	Thr
30.	588	Glu	Asp	Asp	Asp	Asp	Asp	Asp	Asp
31.	637	Ile	Val	Val	Val	Val	Val	Val	Val
32.	643	Lys	Lys	Lys	Lys	Lys	Lys	Glu	Glu
33.	661	Thr	Ser	Ser	Ser	Ser	Ser	Ser	Ser
34.	684	His	Tyr	Tyr	His	His	His	His	His
35.	706	Ile	His	His	His	His	His	His	His
36.	754	Lys	Arg	Arg	Lys	Lys	Lys	Arg	Arg
37.	787	Ala	Thr	Ala	Ala	Ala	Ala	Ala	Ala
38.	830	Met	Glu	Glu	Glu	Glu	Glu	Glu	Glu
39.	860	Ser	Ala	Ala	Thr	Thr	Thr	Thr	Thr
40.	878	Val	Ile	Ile	Ile	Ile	Ile	Ile	Ile
41.	883	Asn	Thr	Thr	Thr	Thr	Thr	Thr	Thr

Table S2: Amino acid mutations in structural and non-structural proteins of JEV GIV isolates. All amino acid mutations of JEV GIV were counted based compared P3 strain. The table is designed on the basis of strians and gene structure respectively. Within the GIV, it's divided into two groups, old sublineage and new sublineage, according to the isolated time. And the old sublineage group includes strain JKT6468 and VN_ 113. New sublineage group is also divided into two groups, Indonesia branch and Australia branch, according to the separation site. Indonesia branch includes the strain JEV/sw/Bali/93/2017, Bali 2019 and 19CxBa-83-Cv, and Australia branch includes the strain JEV/Human/NT_ Tiwi Islands/2021 and JEV/sw-22-00722-11/Qld/2022. The sequence number is discontinuous and only indicates the sequence of mutations in the protein. NS4B has no amino acid mutation, so it isn't taken into account.
