# Supplementary figures and images for: Re-Emerged Genotype IV of Japanese Encephalitis Virus Is the Youngest Virus in Evolution
Source: Viruses. 2023 Feb 24;15(3):626. doi: 10.3390/v15030626 (PMC10054483; doi:10.3390/v15030626)

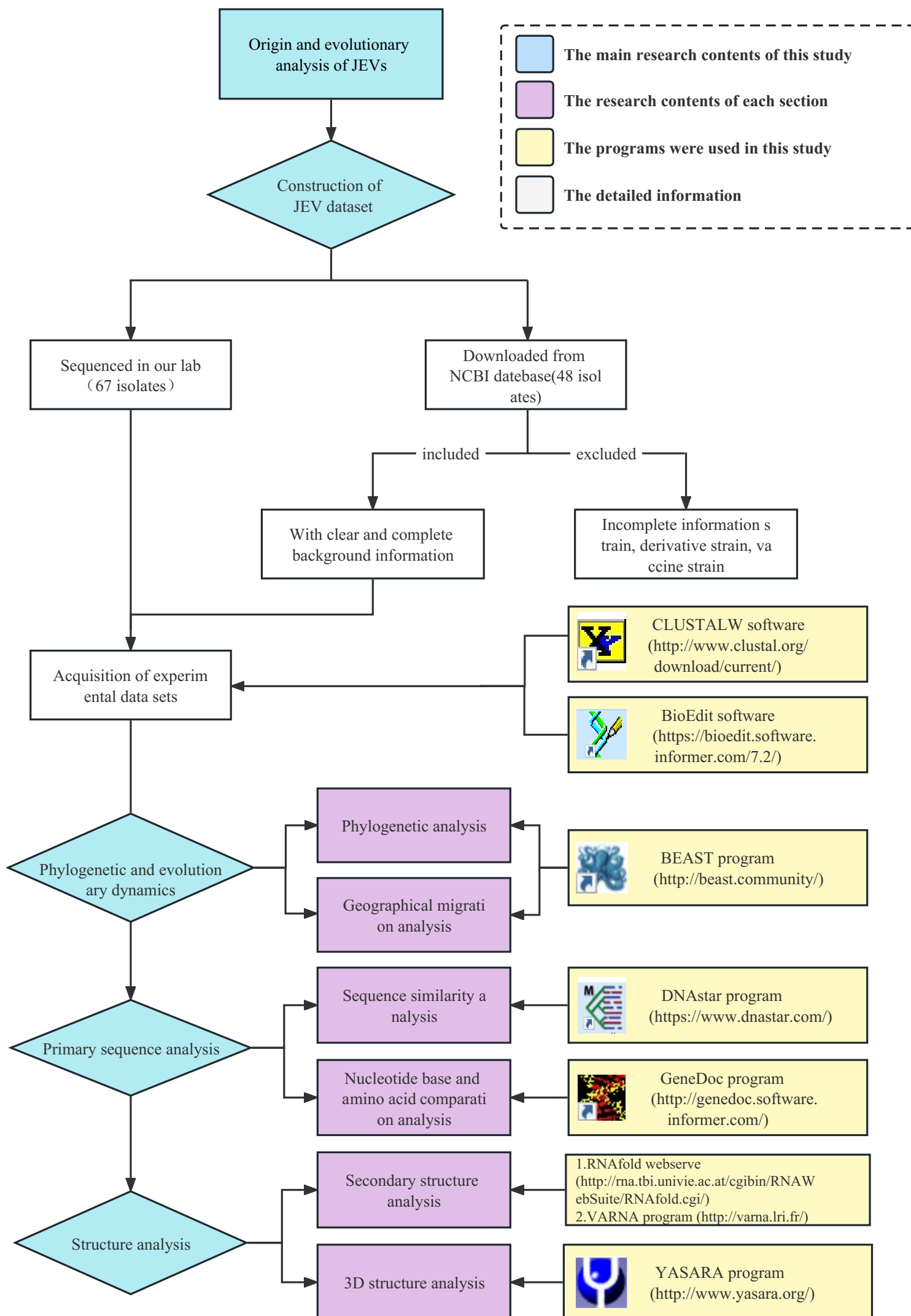

Supplement: Supplementary file 1 [file viruses-15-00626-s001.zip › Figure S1:Analysis pipline.pdf]
