# Supplementary material for: Re-Emerged Genotype IV of Japanese Encephalitis Virus Is the Youngest Virus in Evolution
Source: Viruses. 2023 Feb 24;15(3):626. doi: 10.3390/v15030626 (PMC10054483; doi:10.3390/v15030626)
Supplement: Supplementary file 1 [file viruses-15-00626-s001.zip › Table S1:The average nucleotide and amino acid identities of the JEVs’ whole genome.doc]

Table S1: The average nucleotide and amino acid identities of the JEVs’ whole genome

| Within genotypes (nt %, aa%) | | | | | | | | | | | | | |
| --- | --- | --- | --- | --- | --- | --- | --- | --- | --- | --- | --- | --- | --- |
| Genotype | 5'UTR | C | PrM | E | NS1 | NS2A | NS2B | NS3 | NS4A | NS4B | NS5 | 3'UTR | Complete |
| Ⅰ | 99.0 | 97.8(98.2) | 96.7(98.0) | 96.8(99.2) | 97.6(99.5) | 97.0(98.7) | 96.8(99.5) | 97.3(99.5) | 97.1(99.5) | 97.7(99.7) | 97.2(99.3) | 96.1 | 97.1(99.2) |
| Ⅱ | 100 | 100(100) | 100(100) | 100(100) | 100(100) | 100(100) | 100(100) | 100(100) | 100(100) | 100(100) | 100(100) | 100 | 100(100) |
| Ⅲ | 99.1 | 97.6(98.1) | 97.1(98.5) | 97.3(98.7) | 97.6(99.0) | 97.0(98.7) | 97.4(99.2) | 97.4(99.3) | 97.0(99.2) | 97.0(99.2) | 97.5(99.2) | 96.9 | 97.4(99.0) |
| Ⅳ | **98.3** | **97.3(95.4)** | **95.3(96.0)** | **96.6(98.6)** | **97.5(99.6)** | **97.0(99.3)** | **97.2(99.8)** | **96.4(99.1)** | **96.7(99.8)** | **96.2(100)** | **96.8(99.2)** | **91.5** | **96.4(98.9)** |
| Ⅴ | 98.8 | 95.4(95.8) | 96.2(99.7) | 93.9 (99.2) | 94.3(99.0) | 93.3(99.2) | 94.1(99.3) | 94.4(99.1) | 92.9(97.7) | 94.2(100) | 94.3(99.0) | 95.4 | 94.3(98.9) |

Table S1: The average nucleotide and amino acid identities of the JEVs’ whole genome. The table shows the nucleotide and amino acid identities of structural protein, non-structural protein, non-coding region and whole genome of JEV in genotypes. The amino acid identities are shown in brackets. There is only one isolate of GⅡ, which cannot display more information. GⅣ JEV is our most concerned in this study, which is marked by bold.
