# Supplementary material for: Re-Emerged Genotype IV of Japanese Encephalitis Virus Is the Youngest Virus in Evolution
Source: Viruses. 2023 Feb 24;15(3):626. doi: 10.3390/v15030626 (PMC10054483; doi:10.3390/v15030626)
Supplement: Supplementary file 1 [file viruses-15-00626-s001.zip › Table S2: Amino acid mutations in structural and non-structural proteins of JEV GIV isolates.doc]

**Table S2:** Amino acid mutations in structural and non-structural proteins of JEV GⅣ isolates

|  | | | | **GⅣ** | | | | | | |
| --- | --- | --- | --- | --- | --- | --- | --- | --- | --- | --- |
| **No.** | **Protein** | **Position of mutation** | **Standard strain** | **Old sublineage** | | **Emerging sublineage** | | | | |
|  |  |  | P3 | Jkt6468 | VN_113 | Indonesia branch | | | Australia branch | |
|  |  |  |  |  |  | Bali 93 | Bali-2019 | CxBa_83-CV | Nt-tiwi | Sw-22-0072 |
| 1. | C | 6 | Gly | Arg | Gly | Gly | Gly | Gly | Gly | Gly |
| 2. |  | 10 | Lys | Ile | Lys | Lys | Lys | Lys | Lys | Lys |
| 3. |  | 15 | Asn | Tyr | Asn | Asn | Asn | Asn | Asn | Asn |
| 4. |  | 41 | Arg | Ile | Arg | Arg | Arg | Arg | Arg | Arg |
| 5. |  | 50 | Phe | Leu | Leu | Leu | Leu | Leu | Leu | Leu |
| 6. |  | 52 | Thr | Ser | Ala | Ala | Ala | Ala | Ala | Ala |
| 7. |  | 60 | Ala | Ser | Ala | Ala | Ala | Ala | Ala | Ala |
| 8. |  | 75 | Ser | Ser | Ser | Gly | Gly | Gly | Gly | Gly |
| 9. |  | 109 | Glu | Gly | Gly | Gly | Gly | Gly | Gly | Gly |
| 10. |  | 111 | Ser | Thr | Thr | Thr | Thr | Thr | Thr | Thr |
| 11. |  | 112 | Ile | Thr | Thr | Val | Val | Val | Val | Val |
| 12. |  | 113 | Met | Leu | Leu | Leu | Leu | Leu | Leu | Leu |
| 13. |  | 115 | Leu | Phe | Phe | Leu | Leu | Leu | Leu | Leu |
| 14. |  | 116 | Ala | Met | Met | Met | Met | Met | Met | Met |
| 15. |  | 119 | Ala | Thr | Thr | Thr | Thr | Thr | Thr | Thr |
| 16. |  | 120 | Ala | Ile | Ile | Ile | Ile | Ile | Ile | Ile |
| 17. |  | 121 | Val | Ala | Ala | Ala | Ala | Ala | Ala | Ala |
| 18. |  | 122 | Ile | Ala | Ala | Gly | Gly | Gly | Ala | Ala |
| 19. |  | 123 | Ala | Val | Val | Val | Ile | Val | Val | Val |
| 20. |  | 124 | Cys | Cys | Cys | Ser | Ser | Ser | Ser | Ser |
| 21. |  | 125 | Ala | Val | Val | Val | Val | Val | Val | Val |
| 1. | PrM | 1 | Met | Leu | Leu | Leu | Leu | Leu | Leu | Leu |
| 2. |  | 2 | Arg | Lys | Lys | Lys | Lys | Lys | Lys | Lys |
| 3. |  | 13 | Thr | Ala | Ala | Thr | Thr | Thr | Thr | Thr |
| 4. |  | 14 | Ile | Val | Val | Ile | Ile | Ile | Ile | Ile |
| 5. |  | 44 | Met | Met | Met | Met | Met | Met | Thr | Thr |
| 6. |  | 55 | Lys | Lys | Lys | Arg | Arg | Arg | Arg | Arg |
| 7. |  | 57 | Thr | Thr | Ala | Ala | Ala | Ala | Ala | Ala |
| 8. |  | 58 | Met | Pro | Pro | Pro | Pro | Pro | Pro | Pro |
| 9. |  | 63 | Glu | Gln | Glu | Glu | Glu | Glu | Glu | Glu |
| 10. |  | 71 | Asn | His | Asn | Asn | Asn | Asn | Asn | Asn |
| 11. |  | 73 | Glu | Glu | Asp | Asp | Asp | Asp | Asp | Asp |
| 12. |  | 85 | Arg | Ser | Arg | Arg | Arg | Arg | Arg | Arg |
| 13. |  | 88 | Lys | Gln | Lys | Lys | Lys | Lys | Lys | Lys |
| 14. |  | 89 | Arg | Thr | Arg | Arg | Arg | Arg | Arg | Arg |
| 15. |  | 97 | Gln | His | Gln | Gln | Gln | Gln | Gln | Gln |
| 16. |  | 107 | Lys | Lys | Lys | Lys | Lys | Arg | Lys | Lys |
| 17. |  | 113 | Asp | Asn | Asp | Asp | Asp | Asp | Asp | Asp |
| 18. |  | 122 | Met | Val | Met | Met | Met | Met | Met | Met |
| 19. |  | 130 | Ile | Val | Ile | Ile | Ile | Ile | Ile | Ile |
| 20. |  | 139 | Ala | Ala | Val | Val | Val | Val | Val | Val |
| 21. |  | 140 | Val | Val | Ala | Ala | Ala | Ala | Ala | Ala |
| 22. |  | 149 | Asn | Ser | Ser | Ser | Ser | Ser | Ser | Ser |
| 23. |  | 151 | Gln | Pro | Gln | Gln | Gln | Gln | Gln | Gln |
| 24. |  | 153 | Val | Val | Val | Val | Val | Val | Met | Met |
| 1. | E | 15 | Ala | Val | Val | Val | Val | Val | Val | Val |
| 2. |  | 36 | Asn | Asn | Asn | His | His | His | His | His |
| 3. |  | 38 | Lys | Arg | Arg | Arg | Arg | Arg | Arg | Arg |
| 4. |  | 46 | Thr | Ile | Ile | Ile | Ile | Val | Ile | Ile |
| 5. |  | 76 | Met | Thr | Thr | Thr | Thr | Thr | Thr | Thr |
| 6. |  | 83 | Glu | Glu | Glu | Asp | Asp | Asp | Glu | Glu |
| 7. |  | 89 | Ser | Ser | Ser | Asn | Asn | Asn | Ser | Ser |
| 8. |  | 126 | Ile | Thr | Thr | Thr | Thr | Thr | Ile | Ile |
| 9. |  | 128 | Arg | Lys | Lys | Lys | Lys | Lys | Lys | Lys |
| 10. |  | 129 | Ala | Thr | Thr | Thr | Thr | Thr | Thr | Thr |
| 11. |  | 141 | Ile | Ile | Ile | Ile | Ile | Ile | Val | Val |
| 12. |  | 156 | Ser | Thr | Thr | Thr | Thr | Thr | Thr | Thr |
| 13. |  | 159 | Val | Ile | Ile | Ile | Ile | Ile | Ile | Ile |
| 14. |  | 169 | Val | Ile | Ile | Ile | Ile | Ile | Ile | Ile |
| 15. |  | 194 | Ser | Ser | Ser | Ser | Ser | Ser | Asn | Asn |
| 16. |  | 209 | Arg | Lys | Lys | Lys | Lys | Lys | Lys | Lys |
| 17. |  | 227 | Pro | Ser | Ser | Ser | Ser | Ser | Ser | Ser |
| 18. |  | 228 | Pro | Ser | Ser | Ser | Ser | Ala | Ser | Ser |
| 19. |  | 260 | Gly | Arg | Gly | Gly | Gly | Gly | Gly | Gly |
| 20. |  | 261 | Gly | Ala | Ala | Ala | Ala | Ala | Ala | Ala |
| 21. |  | 295 | Ala | Ala | Ala | Thr | Thr | Thr | Thr | Thr |
| 22. |  | 306 | Gly | Glu | Glu | Glu | Glu | Glu | Glu | Glu |
| 23. |  | 327 | Ser | Leu | Leu | Gln | Gln | Gln | Gln | Gln |
| 24. |  | 351 | Ala | Val | Val | Val | Val | Val | Val | Val |
| 25. |  | 366 | Ala | Ser | Ser | Ser | Ser | Ser | Ser | Ser |
| 26. |  | 370 | Lys | Gln | Lys | Lys | Lys | Lys | Lys | Lys |
| 27. |  | 388 | Glu | Gly | Gly | Gly | Gly | Gly | Gly | Gly |
| 28. |  | 399 | Ala | Pro | Pro | Pro | Pro | Pro | Pro | Pro |
| 29. |  | 408 | Leu | Ser | Ser | Ser | Ser | Ser | Ser | Ser |
| 30. |  | 466 | Ala | Val | Val | Ala | Ala | Ala | Ala | Ala |
| 31. |  | 473 | Val | Ile | Ile | Val | Val | Val | Ile | Ile |
| 32. |  | 482 | Leu | Met | Met | Met | Met | Met | Val | Val |
| 33. |  | 486 | Ala | Val | Val | Val | Val | Val | Val | Val |
| 34. |  | 490 | Val | Thr | Thr | Thr | Thr | Thr | Thr | Thr |
| 35. |  | 492 | Val | Leu | Leu | Leu | Leu | Leu | Leu | Leu |
| 1． | NS1 | 51 | Lys | Met | Met | Met | Met | Met | Met | Met |
| 2． |  | 54 | Val | Ile | Ile | Ile | Ile | Ile | Ile | Ile |
| 3． |  | 95 | Ser | Ser | Ser | Ser | Ser | Ser | Pro | Pro |
| 4． |  | 175 | Ser | Asn | Asn | Asn | Asn | Asn | Asn | Asn |
| 5． |  | 182 | Ala | Thr | Thr | Thr | Thr | Thr | Thr | Thr |
| 6． |  | 188 | Val | Ile | Ile | Ile | Ile | Ile | Ile | Ile |
| 7． |  | 205 | Arg | His | His | His | His | His | His | His |
| 8． |  | 206 | Tyr | Leu | Leu | Leu | Leu | Leu | Leu | Leu |
| 9． |  | 220 | Val | Ile | Ile | Ile | Ile | Ile | Ile | Ile |
| 10． |  | 271 | Asn | Asp | Asp | Asp | Asp | Asp | Asp | Asp |
| 11． |  | 317 | Ser | Thr | Thr | Thr | Thr | Thr | Thr | Thr |
| 12． |  | 326 | Glu | Gly | Gly | Gly | Gly | Gly | Gly | Gly |
| 13． |  | 327 | Asn | Ser | Ser | Ser | Ser | Ser | Ser | Ser |
| 14． |  | 335 | Ile | Val | Val | Val | Val | Val | Val | Val |
| 15． |  | 339 | Arg | Lys | Lys | Lys | Lys | Lys | Lys | Lys |
| 16． |  | 343 | Thr | Ala | Ala | Ala | Ala | Ala | Ala | Ala |
| 17． |  | 350 | Val | Ala | Ala | Val | Val | Val | Val | Val |
| 18． |  | 354 | Asn | Ser | Ser | Ser | Ser | Ser | Ser | Ser |
| 19． |  | 358 | Val | Ile | Ile | Ile | Ile | Ile | Ile | Ile |
| 20． |  | 386 | Ile | Val | Val | Val | Val | Val | Val | Val |
| 21． |  | 407 | Ala | Ala | Ala | Val | Ala | Ala | Ala | Ala |
| 1. | NS2A | 7 | Ser | Asn | Asn | Asn | Asn | Asn | Asn | Asn |
| 2. |  | 29 | Met | Ala | Ala | Ala | Ala | Ala | Ala | Ala |
| 3. |  | 56 | Val | Ala | Ala | Ala | Ala | Ala | Ala | Ala |
| 4. |  | 65 | Ile | Met | Met | Met | Met | Met | Met | Met |
| 5. |  | 71 | Ile | Ile | Ile | Ile | Ile | Ile | Thr | Ile |
| 6. |  | 76 | Val | Ile | Ile | Ile | Ile | Ile | Ile | Ile |
| 7. |  | 77 | Arg | Lys | Lys | Lys | Lys | Lys | Lys | Lys |
| 8. |  | 86 | Ser | Thr | Thr | Thr | Thr | Thr | Thr | Thr |
| 9. |  | 87 | Val | Ile | Ile | Ile | Ile | Ile | Ile | Ile |
| 10. |  | 91 | Val | Leu | Leu | Leu | Leu | Leu | Leu | Leu |
| 11. |  | 97 | Thr | Ala | Ala | Ala | Ala | Val | Ala | Ala |
| 12. |  | 113 | Val | Ile | Ile | Ile | Ile | Ile | Ile | Ile |
| 13. |  | 116 | Ile | Val | Val | Val | Val | Val | Val | Val |
| 14. |  | 121 | His | Gln | Gln | Gln | Gln | Gln | Gln | Gln |
| 15. |  | 124 | Lys | Arg | Arg | Arg | Arg | Arg | Arg | Arg |
| 16. |  | 125 | Lys | Arg | Arg | Lys | Lys | Lys | Arg | Arg |
| 1. | NS2B | 56 | Gln | Arg | Arg | Arg | Arg | Arg | Arg | Arg |
| 2. |  | 88 | Ile | Ile | Ile | Ile | Ile | Val | Ile | Ile |
| 1. | NS3 | 14 | Ser | Ala | Ala | Thr | Thr | Thr | Thr | Thr |
| 2. |  | 44 | Asn | Ser | Ser | Gly | Gly | Gly | Gly | Gly |
| 3. |  | 92 | Thr | Thr | Thr | Lys | Lys | Lys | Lys | Lys |
| 4. |  | 107 | Val | Thr | Thr | Ile | Ile | Ile | Ile | Ile |
| 5. |  | 117 | Arg | Cys | Cys | Cys | Cys | Cys | Cys | Cys |
| 6. |  | 169 | Asp | Asp | Asp | Glu | Glu | Glu | Glu | Glu |
| 7. |  | 175 | Val | Ile | Ile | Ile | Ile | Ile | Ile | Ile |
| 8. |  | 180 | Thr | Asn | Asn | Asn | Asn | Asn | Asn | Asn |
| 9. |  | 182 | Asn | Ser | Ser | Ser | Ser | Ser | Ser | Ser |
| 10. |  | 185 | Arg | Lys | Lys | Lys | Lys | Lys | Lys | Lys |
| 11. |  | 210 | Lys | Arg | Arg | Arg | Arg | Arg | Arg | Arg |
| 12. |  | 253 | Gln | Gln | Gln | Lys | Gln | Gln | Gln | Gln |
| 13. |  | 294 | Ser | Gly | Ser | Ser | Ser | Ser | Ser | Ser |
| 14. |  | 340 | Ile | Val | Val | Ile | Ile | Ile | Val | Val |
| 15. |  | 374 | Met | Val | Val | Val | Val | Val | Val | Val |
| 16. |  | 382 | Lys | Lys | Lys | Arg | Arg | Arg | Arg | Arg |
| 17. |  | 436 | Glu | Glu | Glu | Glu | Glu | Glu | Gly | Glu |
| 18. |  | 492 | Lys | Lys | Lys | Lys | Lys | Lys | Lys | Arg |
| 19. |  | 495 | Met | Leu | Leu | Leu | Leu | Leu | Leu | Leu |
| 20. |  | 518 | Ser | Phe | Phe | Phe | Phe | Phe | Phe | Phe |
| 21. |  | 586 | Met | Thr | Thr | Met | Met | Met | Met | Met |
| 22. |  | 591 | Ile | Val | Val | Ile | Ile | Ile | Ile | Ile |
| 1. | NS4A | 3 | Val | Ile | Ile | Ile | Ile | Ile | Ile | Ile |
| 2. |  | 5 | Phe | Phe | Phe | Phe | Phe | Phe | Leu | Leu |
| 3. |  | 17 | Met | Ala | Ala | Ala | Ala | Ala | Ala | Ala |
| 4. |  | 35 | Lys | Arg | Arg | Arg | Arg | Arg | Arg | Arg |
| 5. |  | 61 | Thr | Ala | Ala | Ala | Ala | Ala | Ala | Ala |
| 6. |  | 72 | Met | Lys | Met | Met | Met | Met | Met | Met |
| 7. |  | 88 | Thr | Val | Val | Val | Val | Val | Val | Val |
| 8. |  | 100 | Pro | Ser | Ser | Ser | Ser | Ser | Ser | Ser |
| 9. |  | 160 | Thr | Ala | Ala | Ala | Ala | Ala | Ala | Ala |
| 10. |  | 161 | Gln | Pro | Pro | Pro | Pro | Pro | Pro | Pro |
| 11. |  | 162 | Ala | Val | Val | Val | Val | Val | Val | Val |
| 12. |  | 165 | Leu | Met | Met | Met | Met | Met | Met | Met |
| 13. |  | 180 | Ser | Gly | Gly | Gly | Gly | Gly | Gly | Gly |
| 14. |  | 208 | Leu | Ile | Ile | Ile | Ile | Ile | Ile | Ile |
| 15. |  | 264 | Met | Val | Val | Val | Val | Val | Val | Val |
| 16. |  | 267 | Ala | Thr | Thr | Thr | Thr | Thr | Thr | Thr |
| 1. | NS5 | 15 | Lys | Arg | Arg | Arg | Arg | Arg | Arg | Arg |
| 2. |  | 33 | Ile | Ile | Ile | Ile | Ile | Ile | Thr | Thr |
| 3. |  | 49 | Ile | Lys | Lys | Lys | Lys | Lys | Lys | Lys |
| 4. |  | 72 | Ser | Ser | Ser | Ser | Ser | Ser | Thr | Thr |
| 5. |  | 78 | Ile | Val | Val | Ile | Ile | Ile | Ile | Ile |
| 6. |  | 135 | Lys | Arg | Arg | Arg | Arg | Arg | Arg | Arg |
| 7. |  | 144 | Phe | Leu | Leu | Leu | Leu | Leu | Leu | Leu |
| 8. |  | 275 | Asn | Asp | Asp | Asp | Asp | Asp | Asp | Asp |
| 9. |  | 277 | Glu | Gly | Gly | Gly | Gly | Gly | Gly | Gly |
| 10. |  | 283 | Ile | Ile | Ile | Val | Ile | Ile | Ile | Ile |
| 11. |  | 284 | Gln | Gln | Gln | Gln | Gln | Gln | Glu | Glu |
| 12. |  | 292 | Thr | Thr | Thr | Thr | Thr | Ala | Thr | Thr |
| 13. |  | 296 | Lys | Arg | Arg | Arg | Arg | Arg | Arg | Arg |
| 14. |  | 370 | Ala | Pro | Pro | Ser | Ser | Ser | Ser | Ser |
| 15. |  | 372 | Ala | Val | Val | Val | Val | Val | Val | Val |
| 16. |  | 386 | Tyr | Tyr | Tyr | Tyr | Tyr | Tyr | His | His |
| 17. |  | 390 | Glu | Glu | Glu | Glu | Glu | Glu | Glu | Glu |
| 18. |  | 429 | Asp | Gly | Gly | Asp | Asp | Asp | Gly | Gly |
| 19. |  | 439 | Glu | Val | Val | Val | Val | Val | Val | Val |
| 20. |  | 452 | Arg | Cys | Cys | Cys | Cys | Cys | Cys | Cys |
| 21. |  | 453 | Ile | Val | Val | Val | Val | Val | Val | Val |
| 22. |  | 455 | Asn | His | Asn | Asn | Asn | Asn | Asn | Asn |
| 23. |  | 503 | Glu | Arg | Arg | Arg | Arg | Arg | Arg | Arg |
| 24. |  | 526 | Gly | Arg | Arg | Arg | Arg | Arg | Arg | Arg |
| 25. |  | 528 | Gln | Glu | Glu | Glu | Glu | Glu | Glu | Glu |
| 26. |  | 546 | Arg | Lys | Lys | Lys | Lys | Lys | Lys | Lys |
| 27. |  | 576 | Arg | Lys | Lys | Lys | Lys | Lys | Lys | Lys |
| 28. |  | 586 | Ala | Ser | Ser | Ser | Ser | Ser | Ser | Ser |
| 29. |  | 587 | Ala | Thr | Thr | Thr | Thr | Thr | Thr | Thr |
| 30. |  | 588 | Glu | Asp | Asp | Asp | Asp | Asp | Asp | Asp |
| 31. |  | 637 | Ile | Val | Val | Val | Val | Val | Val | Val |
| 32. |  | 643 | Lys | Lys | Lys | Lys | Lys | Lys | Glu | Glu |
| 33. |  | 661 | Thr | Ser | Ser | Ser | Ser | Ser | Ser | Ser |
| 34. |  | 684 | His | Tyr | Tyr | His | His | His | His | His |
| 35. |  | 706 | Ile | His | His | His | His | His | His | His |
| 36. |  | 754 | Lys | Arg | Arg | Lys | Lys | Lys | Arg | Arg |
| 37. |  | 787 | Ala | Thr | Ala | Ala | Ala | Ala | Ala | Ala |
| 38. |  | 830 | Met | Glu | Glu | Glu | Glu | Glu | Glu | Glu |
| 39. |  | 860 | Ser | Ala | Ala | Thr | Thr | Thr | Thr | Thr |
| 40. |  | 878 | Val | Ile | Ile | Ile | Ile | Ile | Ile | Ile |
| 41. |  | 883 | Asn | Thr | Thr | Thr | Thr | Thr | Thr | Thr |

**Table S2:** Amino acid mutations in structural and non-structural proteins of JEV GⅣ isolates. All amino acid mutations of JEV GⅣ were counted based compared P3 strain. The table is designed on the basis of strians and gene structure respectively. Within the GⅣ, it’s divided into two groups, old sublineage and new sublineage, according to the isolated time. And the old sublineage group includes strain JKT6468 and VN_ 113. New sublineage group is also divided into two groups, Indonesia branch and Australia branch, according to the separation site. Indonesia branch includes the strain JEV/sw/Bali/93/2017, Bali 2019 and 19CxBa-83-Cv, and Australia branch includes the strain JEV/Human/NT_ Tiwi Islands/2021 and JEV/sw-22-00722-11/Qld/2022. The sequence number is discontinuous and only indicates the sequence of mutations in the protein. NS4B has no amino acid mutation, so it isn’t taken into account.
